# Maternal fresh moringa leaf consumption and its association with birth weight in southern Ethiopia: A prospective cohort study

**DOI:** 10.1002/fsn3.4453

**Published:** 2024-09-15

**Authors:** Zeritu Dewana Derbo, Gurmesa Tura Debelew

**Affiliations:** ^1^ Department of Midwifery Arba Minch Health Science College Arba Minch Ethiopia; ^2^ Population and Family Health, Institute of Health Jimma University Jimma Ethiopia

**Keywords:** birth weight, consumption, maternal, moringa leaf, pregnancy, southern Ethiopia

## Abstract

Birth weight is an indicator of neonatal survival and development; however, poor nutrient intake during pregnancy is a primary contributor to low birth weight. Moringa is a multipurpose tree high in macro‐ and micronutrients. There is insufficient evidence on the relationship between fresh moringa leaf consumption during pregnancy and birth weight. The purpose of this study was to determine the association between maternal fresh moringa leaf consumption on birth weight in southern Ethiopia. A community‐based, prospective cohort study design was used with 230 pregnant women who consumed fresh moringa leaves and 230 who did not consume moringa leaves. The mothers were enrolled in their second trimester and monitored until delivery. The structural equation model was used to analyze *β* coefficients with *p*‐values <.05. The mean birth weight of newborns born to mothers who took fresh moringa leaves during pregnancy was 3334.42 g, which was considerably higher than the non‐consumer 3196.73 g (*p* = .008). Consuming it during pregnancy significantly increased birth weight by 115.77 g compared to non‐consumers (*β* = 115.77; SE = 43.03: *p* = .007). The study found that eating fresh moringa leaves during pregnancy increased the birth weight of the newborn. As a result, policymakers and managers of mother and child health programs should strive to promote the use of fresh moringa leaves throughout pregnancy. However, more clinical trials are required to discover the ideal/optimal amount per day and duration of fresh moringa leaves for best outcomes.

## Graphical abstract

The average birth weight of babies born to mothers who consumed fresh moringa leaves during pregnancy was 3334.42 g, notably higher than the 3196.73 g for those who did not consume it (*p* = .008). This dietary choice during pregnancy led to a significant increase in birth weight by 115.77 grams compared to those who did not consume moringa (*β* = 115.77; SE = 43.03; *p* = .007). The study concluded that consuming fresh moringa leaves during pregnancy resulted in a higher birth weight for the newborns.

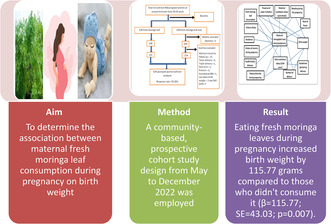

## 1 INTRODUCTION

Birth weight is the body weight of a newborn, and it is a strong predictor of infant survival and development (World Health Organization, 2023; World Health Organization, 2004). Low birth weight (LBW) is defined as a birth weight below 2500 g (Tchamo et al., 2016). In 2020, out of 19.8 million newborns worldwide, about 14.7% were born with low birth weight. Among these, nearly all (95.6%) were in low‐ and middle‐income countries, and one‐third of newborns in Africa were born with low birth weight (World Health Organization, 2023). The highest prevalence of LBW was observed in Sub‐Saharan Africa (14.3%) (Tchamo et al., 2016). It was 13.4% in Burkina Faso, 10.2% in Ghana, 12.1% in Malawi, 15.7% in Senegal, and 10% in Uganda (He et al., 2018).

In Ethiopia, the pooled prevalence of LBW was 14.1%, with the highest prevalence in Tigray 15.4% and the lowest in Addis Ababa 8.7% (Katiso et al., 2020). Factors such as iron‐folate supplementation, meal frequency, minimum dietary diversity, and maternal hemoglobin levels during pregnancy were common contributors of LBW (Adane et al., 2014; CSA & ICF, 2016; Gebremedhin et al., 2015; Girma et al., 2019; Hirut & Kebebush, 2016; Katiso et al., 2020; Mekie & Taklual, 2019; Seid et al., 2019; Teklehaimanot et al., 2014).

Neonates born with LBW face a high risk of mortality within the first month of life (World Health Organization, 2011), and those who survive face lifelong consequences, including a higher incidence of stunting (DeMaeyer et al., 1989), lower intelligence quotient (IQ) (World Health Organization, 2006), and an increased risk of diabetes, overweight, and non‐communicable diseases in adulthood (Nair et al., 2016; Stangret et al., 2017; World Health Organization, 2023).

A healthy pregnancy can prevent and treat factors contributing to LBW (Gernand et al., 2016), increasing the chances of survival and thriving (World Health Organization, 2023). Focusing on maternal nutrition during pregnancy is crucial to achieve healthy pregnancy (USAID, 2018).

Moringa, a fast‐growing, drought‐resistant tropical tree, is rich in both macro‐ and micronutrients, as evidenced by study done in Ethiopia. The study conducted in Ethiopia indicated that the average household daily consumption of moringa was 15 g and provides the daily recommended intake of 18.2% energy, 24.6% protein, 43.5% iron, and 125% calcium. These suggest that moringa species found in Ethiopia are rich in nutrients (Abuye et al., 2003; Tekle et al., 2015).

Studies evidenced that moringa is safe and provides nutritional and health benefit for pregnant women. Moreover, it reduces adverse pregnancy outcomes such as LBW and premature birth (Hadju, Dassir, et al., 2020; Muis et al., 2014; Nurdin, Imam, et al., 2018; Singh & Chaturvedi, 2021). Also, another study revealed that no newborns with LBW were reported among women who consumed moringa leaf extract during pregnancy (Iskandar et al., 2015). And a 353 g increase in birth weight was reported among women who supplemented with moringa leaf powder compared to those who supplemented with iron along with folic acid (IFA) (Hadju, Marks, et al., 2020).

Previous studies conducted in Ethiopia have focused on the general population (Terfassa & Negeyo, 2020), the economic value of moringa (Tafesse et al., 2020), and its effect on hemoglobin levels (Derbo & Debelew, 2023). Although fresh moringa leaves are accessible and offer superior macro‐ and micronutrients, there was no evidence regarding their impact on newborn birth weight in Ethiopia. Our research, however, specifically investigates how consuming fresh moringa leaves during pregnancy influences birth weight. Thus, this study aimed to assess the effects of consuming fresh moringa leaves on birth weight in southern Ethiopia.

## 2 MATERIALS AND METHODS

### 2.1 Study setting, period, and design

The study was conducted in Arba Minch zuria and Chencha district of Gamo Zone, south Ethiopia. The main towns of Arba Minch Zuria and Chencha districts are 434 and 443 km South of Addis Ababa, the capital city of Ethiopia, respectively. The expected number of pregnant women in the study area was 12,214 in the year 2021/2022 (Derbo & Debelew, 2023). Fresh moringa leaves are commonly prepared and consumed in the study area in the form of “Kurkufa,” “Fosossie” (directly cooking a corn flour with the leaves), “Kita” (a flat bread locally prepared from various cereals including corn, wheat, barley, or “Teff”), and “Haleko” (directly cooking the moringa leaves in a separate dish‐like spinach and/or in the form of soup) (Derbo & Debelew, 2024). A community‐based, prospective cohort study design from May to December 2022 was employed.

### 2.2 Population, recruitment, and follow‐up

Participant recruitment occurred at three different points: twice during pregnancy and once postpartum. Pregnant women between 20 and 26 weeks gestation were recruited using a list of potential participants from family folders at health posts. Participants were selected proportionally from each kebele within the districts based on the number of pregnant women. Initial home visits were conducted to obtain signed informed consent to participate in the study and gather data on socioeconomic status, demographics, obstetric and health details, dietary intake, and moringa consumption practices. Maternal hemoglobin and MUAC measurements were also taken. Gestational age was calculated using the first day of the last menstrual period (LMP) and the date of data collection, or estimated using tape fundus height measurement if LMP was unknown.

Baseline data were collected from 623 pregnant women. Among these, 230 women who consumed fresh moringa leaves at least 4 days a week in the month before baseline data collection were classified as consumers, while another 230 who had never consumed fresh moringa leaves were classified as non‐consumers. Initially, 460 participants were enrolled in the cohort from May to June 2022 through a house‐to‐house survey.

Two months after baseline data collection, 460 women from the cohort were assessed through a house‐to‐house survey for midline data. Three abortions were recorded—two from the consumer group and one from the non‐consumer group—resulting in 457 eligible participants for follow‐up. At midline, 228 women who continued to consume fresh moringa leaves at least 4 days a week and 229 who never consumed moringa leaves remained in the study.

**FIGURE 1:**
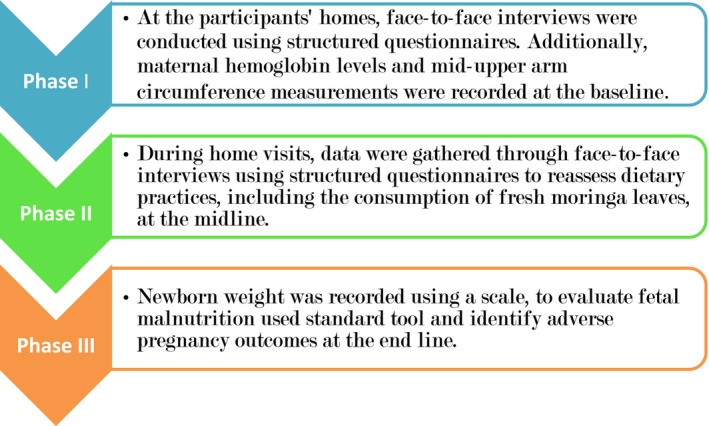
Flowchart of data collection procedures.

At the end of the study, after delivery, data were collected from the remaining 457 women. However, two women had twins, one had triplets, one had a post‐term delivery, three had preterm deliveries, one had gestational diabetes mellitus, two did not have birth weights recorded, three experienced stillbirths, and 15 were lost to follow‐up due to relocation or delivery at a family home. Consequently, 31 participants were excluded, leaving 429 mother–infant pairs for analysis (see Figure 1).

### 2.3 Sample size determination

The sample size was determined using STATA version 14 to compare two independent means. With a 95% confidence interval, a precision of 4%, and 80% power, and considering a mean birth weight of 3360 g with a standard deviation of 870 g (Desta et al., 2019), alongside an anticipated 10% increase in birth weight among those consuming moringa leaves (Hadju, Dassir, et al., 2020; Zerfu et al., 2016), the initial calculated sample size was 91 per group, assuming a 1:1 ratio of consumed to not consumed. To account for potential errors due to multistage cluster sampling, a design effect of two was applied. Additionally, considering a 25% potential loss to follow‐up, the final sample size was 455 and adjusted to 228 participants per group. One of the intermediate and exogenous variables was hemoglobin level of pregnant women. For the purpose of examining the effect of moringa on hemoglobin levels, we used the sample size of 460, comprising 230 pregnant women who consumed fresh moringa leaves at least 4 days per week in the month preceding baseline data collection, and 230 who had never consumed moringa leaves (Derbo & Debelew, 2023), and due to this we were used large sample size 460 (230 consumer and 230 not consumed) participants.

### 2.4 Sampling technique

A multistage sampling method was employed. Twenty kebeles (ten from Arba Minch Zuria and ten from the Chencha district) were selected using lottery methods. Then, eligible pregnant women were identified, and the sample was allocated proportionally to each kebele based on the number of eligible pregnant women. Finally, the required sample was selected using computer‐generated simple random sampling techniques.

### 2.5 Study variable

The study focused on birth weight as the primary endogenous variable. Intermediate variables included maternal hemoglobin levels during pregnancy, and the latent intermediate variable was the newborn's nutritional status, measured by the CAN score. Observed exogenous variables encompassed factors such as the consumption of fresh moringa leaves, place of residence, upper arm circumference during pregnancy, history of anemia, history of pica during pregnancy, gestational age at delivery, number of deliveries, presence of children under 5 years old, and the head of the household. Dietary diversity was considered the latent exogenous variable.

### 2.6 Data collection tools and procedures

Ten experienced nurses and two public health professionals were used to collect and supervise data collection, respectively. Two days of training were conducted for data collectors and supervisors. Three laboratory technicians measured hemoglobin level.

Data collection occurred in three phases: the first two during pregnancy and the final within 48 h postpartum. Baseline data were gathered in the second trimester (20–26 weeks of gestation) at participants' homes through face‐to‐face interviews using structured questionnaires. The collected data included the following: Socioeconomic and Demographic Factors: Mother's age, place of residence, education and occupation of both the mother and her husband, family wealth status, marital status, family size, and head of household; Health and Obstetric Factors: Age at first pregnancy, number of pregnancies, number of children under 5, history of abortion, pregnancy status, presence of nausea or vomiting, health insurance status, contraceptive use, distance to the nearest health center, and antenatal care (ANC) attendance; Nutritional Factors: Meal frequency per day, changes in dietary intake, dietary diversity, food aversions, nutritional counseling, and consumption of fresh moringa leaves, including frequency and duration of intake per week; and Biochemical and Anthropometric Data: Maternal hemoglobin levels and mid‐upper arm circumference of pregnant women, measured using standard procedures.

In the second phase (midline), conducted 2 months after the baseline, data were collected during the third trimester to re‐assess dietary diversity practices and fresh moringa leaf consumption, as well as to monitor any adverse pregnancy outcomes through home visits.

The third phase (end line) after delivery within 48 h of postnatal period focused on fetal malnutrition using a clinical nutrition assessment score of newborn (Singh & Sood, 2018). Measurement was taken immediately after birth for those delivered in the health institutions, however, for those delivered at home took within the first 24 h with a standard newborn scale (Mehare & Sharew, 2020) and any adverse pregnancy outcome was assessed. Data were collected digitally using the open‐source toolkit Kobo Collect and standard tools (Figure 1).

### 2.7 Definition of term

Pica is the term used to describe having an intense craving for and eating nonfood items, such as soil, clay, ice, ashes, paint chips, wall mud, and other nonfood items (Miller et al., 2018).

### 2.8 Operational definition

Moringa consumers: women who consumed fresh moringa leaves at least 4 days per week in the last 1 month before the baseline data collection date until midline data collection date regardless of amount or frequency were classified as consumers and not consumed ever as non‐consumer.

### 2.9 Measurement

Hemoglobin level, nutrition status, and minimum dietary diversity‐women (MDD‐W) details of the measurement are described in previous paper (Derbo & Debelew, 2023).

Fetal malnutrition was assessed using the CAN score within 48 h of delivery. The CAN score consists of nine superficial and recognizable (hair, cheek, chin, neck, arm, back, buttock, leg, chest, and abdomen) signs of fetal malnutrition (Singh & Sood, 2018; Soundarya et al., 2012). Each of the nine parameters was scored on a scale of 1–4 based on inspection and physical examination of the subcutaneous tissue. After summing the scores, newborn with scores less than 25 were classified as fetal malnourished and those with scores of 25 and more were classified as well‐nourished (Singh & Sood, 2018).

The newborn weight was measured with a standard newborn scale to the nearest 1 g. The scale was adjusted to the zero level before weighing each newborn (Mehare & Sharew, 2020).

### 2.10 Data management and statistical analyses

**FIGURE 2:**
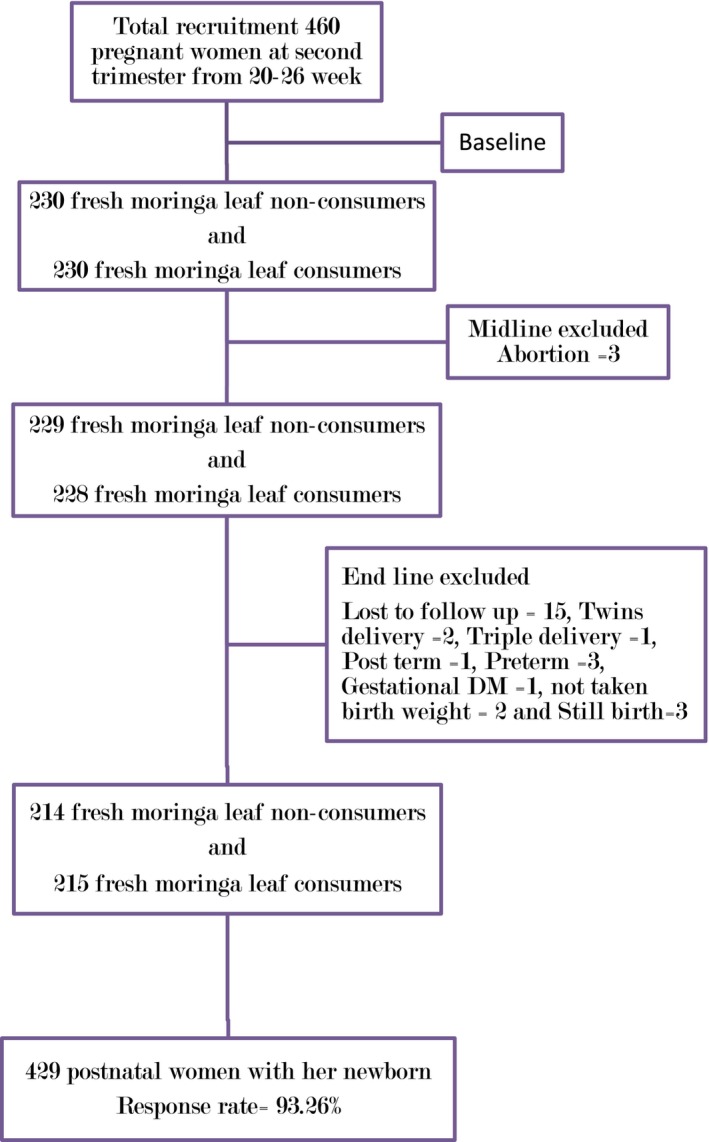
Flowchart of the participants in the longitudinal cohort study.

Data were checked online and approved for its consistency and completeness on a daily basis. After data collection was completed, the data were downloaded and exported to STATA 14 version software for analysis. Data were cleaned, coded, and checked for missing values and outliers before analysis. A total of 31 (6.7%) participants data were excluded from the final analysis due to various reasons (Figure 2).

Descriptive statistics such as frequency, percentages, mean, and standard deviations were used to describe the characteristics of the study participants. Principal component analysis (PCA) was employed to assess both the household wealth status and maternal knowledge regarding the importance of moringa consumption during pregnancy. All assumptions were checked, and items that violated the assumptions were excluded. The sampling adequacy was assessed using Kaiser Mayer Olkin (KMO) of >0.5, and there were significant correlations among items (*p* < .05).

**FIGURE 3:**
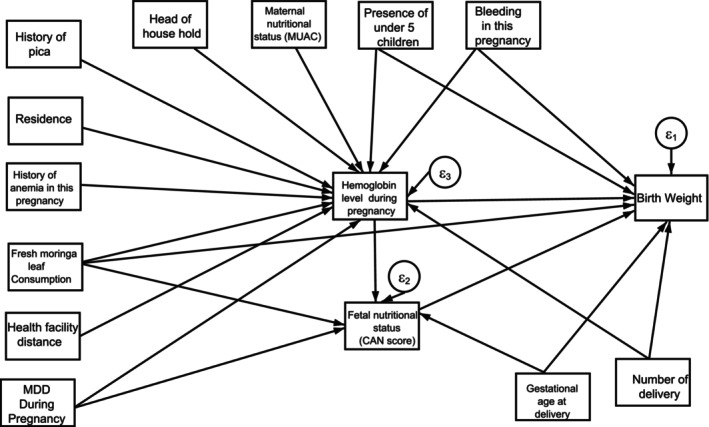
Structural path model of fresh moringa leaf consumption during pregnancy on birth weight.

The mean birth weight was compared using independent *t*‐test. A path diagram was constructed with single arrows representing the causal order between two variables, with the head pointing to the effect and the tail pointing to the cause. The exogenous variable of interest and the resulting birth weight have been developed using directed acyclic graphs. A hypothetical model was built based on previous birth weight theory, literatures, and data driven. Variable with a *p*‐value < .25 in the bivariate analysis was taken into consideration to build path diagram (Figure 3). This was subsequently tested using a statistical modeling technique known as structural equation model (SEM) (Greenland et al., 1999).

Model adequacy was assessed by examining various goodness‐of‐fit indices, including *χ* ^2^/df, RMSEA, CFI, TLI, and SRMR. The model that best fit the data was selected (Table 1).

**TABLE 1:** Fit indices for model.

Indices	Value	Acceptable range
*χ* ^2^/df	13.61/14 = 0.97	<5
*p*‐value	.694	>0.5
RMSEA (90% CI)	0.000 (0.00, 0.035)	Good fit RMSEA ≤0.05
CFI	1.000	Good fit CFI >0.95
TLI	1.023	Good fit TLI >0.95
SRMR	0.017	Good fit SRMR <0.08

Abbreviations: CFI, Comparative fit index; RMSEA, root mean square error of approximation; SRMR, standardized root mean square residual; TLI, Tucker–Lewis index; *χ* ^2^/df, chi‐square divided by the degrees of freedom.

As a result, the hypothetical model was considered appropriate for analyzing the data (Dolatian et al., 2016). STATA version14 statistical software was used to model the direct, indirect, and total effects of intermediate and exogenous variables on birth weight. The *β* coefficient with a 95% CI was used to declare statistical significance at a *p*‐value < .05. The *χ* ^2^/df, RMSEA, CFI, TLI, and SRMR of the final model were all close to or better than the suggested fit (Table 1).

### 2.11 Ethics approval and consent to participate

Ethical approval and clearance were obtained from the Institutional Review Board Institute of Health, Jimma University with reference number (Ref. no. THRPG 1/469/2022). Written permission has been obtained from the Gamo zone health office and respected administrations. Prior to participation, all participants provided written informed consent approved by the Ethics Committee, and all methods were performed in accordance with the declaration of Helsinki ethical principle for medical research involving human subjects.

The data collectors read the consent form to the participants before the interviews to obtain consent, and proceeded after ensuring that they were willing to participate in the study. We aimed to reduce the socially desirable bias by conducting interviews with participants in their own homes. Simultaneously, we ensured the protection of participants' privacy and confidentiality during the entire data collection process.

There was no procedure conducted that could harm pregnant women and her fetus. They have received all the necessary routine nutrition counseling and health services regardless of their participation. Pregnant women with low hemoglobin level were linked with the health extension workers for iron with folic acid supplementation and referred to health facilities for further investigation.

## 3 RESULTS

### 3.1 Socio‐demographic, health, and dietary‐related characteristics of the study participant

At baseline, there was no significant difference between the two groups regarding the head of the household, possession of health insurance, pregnancy status, experience of nausea/vomiting, number of pregnancies, history of abortion, history of anemia, consumption of animal source food (ASF), and maternal nutritional status (*p*‐value > .05) (Table 2).

**TABLE 2:** Socio‐Demographic, health, and dietary‐related characteristics of fresh moringa leaf consumers and non‐consumers women in southern Ethiopia at baseline.

Variable	Non‐consumer no (%)	Consumer no (%)	*p*‐value
Household head			.812
Male	206 (96.26)	206 (95.81)	
Female	8 (3.74)	9(4.19)	
Health insurance			.311
Yes	109 (50.93)	120 (55.81)	
No	105 (49.07)	95 (44.19)	
Number of pregnancy			.232
Primigravida	44 (20.56)	55 (25.58)	
Multigravida	147 (68.69)	145 (67.44)	
Grand multigravida	23 (10.75)	15 (6.98)	
Previous history of abortion			.987
No	193 (90.19)	194 (90.23)	
Yes	21 (9.81)	21 (9.77)	
Pregnancy status			.159
Unplanned	63 (29.44)	77 (35.81)	
Planned	151 (70.56)	138 (64.19)	
Had nausea/vomiting			.729
No	92 (42.99)	96 (44.65)	
Yes	122 (57.01)	119 (55.35)	
Anemia during the current pregnancy			.991
No	202 (94.39)	203 (94.42)	
Yes	12 (5.61)	12 (5.58)	
Maternal nutrition status during pregnancy			.638
Under nursed	73 (34.11)	78 (36.28)	
Well nursed	141 (65.89)	137 (63.72)	
Animal source food (ASF)			.158
Not consume	125 (58.41)	111 (51.63)	
Consume	89 (41.59)	104 (48.37)	

### 3.2 Frequency of consumption of fresh moringa leaves

At the baseline, the average consumption of fresh moringa leaves was 2.31 (SD 0.84) times per day, with a minimum of once per day and a maximum of four times per day. Additionally, the average number of days per week for consumption was 6.42 (SD 1.05), ranging from a minimum of 4 days to a maximum of 7 days per week.

At the midline, the average consumption of fresh moringa leaves was slightly higher at 2.35 (SD 0.80) times per day, with the same range of one to four times per day. The average number of days per week for consumption was 6.44 (SD 1.02), again ranging from 4 to 7 days per week for moringa consumer group. However, individuals in the non‐moringa group never consumed moringa leaves, neither at baseline nor during the midline data collection time.

### 3.3 Fresh moringa leaf consumption during pregnancy on birth weight

The mean birth weight of newborn from mother who consumed fresh moringa leaves during pregnancy was significantly high than non‐consumer, *p* = .008 (Table 3).

**TABLE 3:** The link between fresh moringa leaf consumption during pregnancy and birth weight in southern Ethiopia.

Moringa consumption	Observation	Birth weight mean ± SD	[95% Conf. interval]	*t*‐test	*p*‐value
No	214	3196.73 ± 28.32	3140.90–3252.56	−3.17	0.008
Yes	215	3334.42 ± 32.86	3269.65–3399.19		
Difference		137.69 g	222.99–52.39		

Abbreviation: SD, standard deviation.

Fresh moringa leaf consumption during pregnancy had significant indirect and total effect on birth weight *β* = 90.97, *p*‐value = .001 and *β* = 115.77, *p*‐value = .007, respectively (Table 4).

**TABLE 4:** Direct, indirect, and total effect of maternal fresh moringa leaf consumption during pregnancy and other variables on birth weight.

Outcome	Direct effect *β* (SE) *p*‐value	Indirect effect *β* (SE) *p*‐value	Total effect *β* (SE) *p*‐value
Birth weight in gram			
Fresh moringa leaf consumption during the current pregnancy	24.79 (38.21) *p* = .516	90.97 (25.43) *p* = .001	115.77 (43.03) *p* = .007
Maternal hemoglobin level during the current pregnancy	35.05 (15.40) *p* = .023	24.32 (9.00) *p* = .007	59.38 (17.62) *p* = .001
CAN (clinical assessment of nutritional status) score	53.13 (4.36) *p* = .001	NA	53.13 (4.36) *p* = .001
Residence	NA	21.19 (9.75) *p* = .030	21.19 (9.75) *p* = .030
Maternal MUAC (mid‐upper arm circumference) in cm	NA	11.67 (3.82) *p* = .002	11.67 (3.82) *p* = .002
History of pica during the current pregnancy	NA	−58.04 (24.06) *p* = .016	−58.04 (24.06) *p* = .016
Bleeding encountered during the current pregnancy	−228.48 (98.68) *p* = .021	−44.63 (21.66) *p* = .039	−273.11 (99.43) *p* = .006
The nearest health facility distance	NA	5.01 (1.96) *p* = .011	5.01 (1.96) *p* = .011
Total number of delivery	−0.86 (16.33) *p* = .958	−9.63 (3.99) *p* = .016	−10.49 (16.40) *p* = .522
Gestational age at the current delivery	51.18 (15.14) *p* = .001	30.97(9.14) *p* = .001	82.15(17.31) *p* = .001

Abbreviations: NA, not applicable; *β* (SE), beta coefficient with standard errors.

Hemoglobin level during pregnancy had significant direct (*β* = 35.05, *p*‐value = .023), indirect (*β* = 24.32, *p*‐value = .007), and total (*β* = 59.38, *p* = .001) effect on birth weight. Newborn nutrition status assessed using CAN score had significant direct (*β* = 53.13, *p* = .001) and total (*β* = 53.13, *p* = .001) effect on birth weight. Place of residence had significant indirect effect on birth weight (*β* = 21.19, *p* = .030) and total (*β* = 21.18, *p* = .030) effect on birth weight. Maternal nutritional status assessed using MUAC had significant indirect (*β* = 11.67, *p* = .002) and total (*β* = 11.67, *p* = .002) effect on birth weight. Maternal pica experience during pregnancy had significant indirect (*β* = −58.04, *p* = .016) and total (*β* = −58.04, *p* = .016) effect on birth weight.

Distance to the nearest health facility had indirect (*β* = 5.01, *p* = .011) and total (*β* = 5.01, *p* = .011) effect on birth weight. Bleeding during pregnancy had direct (*β* = −228.48, *p* = .021), indirect (*β* = −44.63, *p* = .039), and total (*β* = −273.11, *p* = .006) effect on birth weight. Number of delivery had indirect (*β* = −9.63, *p* = .016) effect on birth weight. Gestational age at delivery had direct (*β* = 51.18, *p* = .001), indirect (*β* = 30.97, *p* = .001), and total (*β* = 82.16, *p* = .001) effect on birth weight.

## 4 DISCUSSION

In this study, the weight of the newborn was 115.77 g higher among newborn from mothers who consumed fresh moringa leaves compared to non‐consumers. This finding is supported by studies conducted in Central African Republic (Hastuty et al., 2020) and Indonesia (Arundhana et al., 2018; Ningsih, 2018; Rini, 2018). This is due to essential nutrient and antioxidants content of moringa (Abuye et al., 2003; He et al., 2018; Mikore & Mulugeta, 2017; Nurdin, Hadju, et al., 2018) which enhance appetite, restore the digestive system, improve nutrient absorption, enhance placental protein synthesis, facilitate the transfer of nutrients from mother to fetus, and reduce oxidative stress (Ankar & Kumar, 2024; Hallberg et al., 1986; Hussain et al., 2020; Manta‐Vogli et al., 2020; Mekie & Taklual, 2019; Suzuki et al., 2011; Wiley & Gupta, 2024; Wu, 2009) consequently leading to the improvement of the nutritional status of pregnant women and birth weight (Most et al., 2019; Retnakaran et al., 2018; Sartorelli et al., 2021; Tela et al., 2019; Woldeamanuel et al., 2019). Also evidence indicates that consumption of moringa leaf flour, extract, or capsules improves the nutritional status and weight of the participants (Allo et al., 2020; Muis et al., 2014; Nadimin et al., 2019; Renitasari, 2023; Srikanth et al., 2014).

This study found that the weight of the newborn was 11.67 g higher for every 1 cm increase in maternal MUAC. This is supported by studies conducted in Indonesia (Yosefinata et al., 2022). This is due to the fact that enhancing maternal nutrition leads to optimize maternal health and better pregnancy outcomes, eventually resulting in increased birth weight. But a study conducted in Pakistan showed that maternal nutritional status had no significant effect on newborn weight (Sangi et al., 2021).

In the present study, a 1 g/dL increase in maternal hemoglobin level is associated with a 59.38 g increase in birth weight. Similarly, study reported a reduction in the incidence of LBW as maternal hemoglobin level increased (Engidaw et al., 2022; Woldeamanuel et al., 2019). This is the fact that as hemoglobin levels decrease, nutrient and oxygen perfusion also decrease resulting in reduction in fetal energy and nutrient levels (Figueiredo et al., 2018; Fite et al., 2022). Furthermore, there is evidence indicating that anemia stimulates fetal cortisol production which inhibits longitudinal fetal growth. Additionally, anemia heightens the risk of maternal infections that worsen the situation (Allen, 2001).

Newborns born to mothers who experienced pica during pregnancy had a birth weight reduction of 58.04 g compared to their counterparts. This is supported by studies conducted in Pakistan and Iran and systematic review and meta‐analysis (Khazaei et al., 2021; Shah et al., 2022). Pregnancy is a period in which a series of physiological and biochemical changes occur to create a favorable environment for the fetus (Williamson, 2006). Pica, the experience of sudden uncontrollable urges to eat non‐nutritious substances, is one of the common changes that can occur during pregnancy (Fikrie et al., 2022; Sanjari et al., 2023). Women with pica faced a range of health hazards, such as metabolic imbalances, mercury poisoning, hypokalemia, parasitic infections, tooth wear, bowel obstruction, psychotic comorbidities, and deficiencies in iron, zinc, and calcium (Agarwal, 2017). Also pica is an ignored cause of malnutrition in the world (Agarwal, 2017; Francis et al., 2022). Thus, newborns from mothers with pica were at a high risk of low birth weight.

Our study discovered that for each additional kilometer from the nearest health facility, newborn weight increased by 5.01 g. In contrast, a study conducted in Pakistan found that as the distance to healthcare facilities grows, the risk of child malnutrition also rises (Shahid et al., 2022). This discrepancy might be due to our study using distance to the health facility as a continuous variable, whereas the Pakistani study categorized distance into groups. In fact, improved accessibility to health services can enhance healthcare utilization, including continuous maternal care, which may lead to earlier diagnosis and treatment of undernutrition.

In this study, the weight of newborns was 273.11 g lower among neonates born to mothers who faced bleeding during the current pregnancy compared to those who did not. This finding is consistent with studies conducted in Iran and Ethiopia (Hailu & Kebede, 2018; Sadegh et al., 2013). This is due to the fact that bleeding during pregnancy is a common danger sign associated with adverse pregnancy outcomes (Tadese et al., 2021) including low birth weight.

Newborn birth weight from urban dweller was 21.19 g higher than those from rural dweller. This finding is supported by studies conducted in different parts of Ethiopia (Alemu et al., 2019; Mengesha et al., 2017). This is due to differences in accessibility between urban and rural dweller regarding health services, health information, and media exposure. These differences contribute to maternal awareness of healthcare during pregnancy, which in turn affects birth weight.

Our study found that the weight of the newborn was 82.15 g higher for every 1 week increased in gestational age at delivery. This is consistent with study conducted in Northern Ethiopia (Mekonen et al., 2015). Similarly for each increase in parity, the weight of the newborn was reduced by 9.63 g. This finding is supported by studies done in Ethiopia (Alemu et al., 2019; Tafere et al., 2018), but inconsistent with studies done in Sweden and Ethiopia (Lwin et al., 2023; Woldeamanuel et al., 2019). This may be due to socioeconomic status and methodological difference.

The implication of this finding is that encouraging fresh moringa leaf consumption during pregnancy, improving maternal nutrition, preventing anemia, decrease number of delivery through strengthen family planning, minimize risk of bleeding, and educating about the disadvantages of pica would lead to improve birth weight of newborn.

The findings from this study will help advance the Sustainable Development Goals (SDGs) for 2030 by improving health and well‐being, supporting responsible production and consumption, promoting sustainable agriculture, and protecting the environment (Cepal, 2016). They align with the National Reproductive Health Strategy, particularly in the area of maternal, newborn, and adolescent nutrition (3.1.7). Enhancing maternal health and nutrition is crucial for reducing newborn and young child mortality. In turn, efforts to reduce stunting, improve child health, and lower adolescent and overall fertility rates will decrease maternal death risks for future generations (Federal Democratic Republic of Ethiopia Ministry of Health, 2016). Additionally, these insights support the World Health Assembly's nutrition targets for 2012–2025 (Targets, 2025), advocating for the use of local resources to reduce low birth weight (LBW) and, consequently, neonatal morbidity and mortality.

### 4.1 Strengths and limitations

One of the study's strengths is its implementation in a community setting with thorough follow‐up procedures and the use of various data sources. The loss to follow‐up rate was 6.7%, indicating minimal bias. However, the study did not quantify the specific amount of fresh moringa leaves consumed during pregnancy.

### 4.2 Conclusion

Consumption of fresh moringa leaves during pregnancy, along with factors such as hemoglobin levels, fetal and maternal nutritional status, residence, history of pica, bleeding, distance to health facilities, parity, and gestational age at delivery, were identified as independent predictors of birth weight. Health authorities should therefore encourage moringa consumption. However, additional clinical trials are needed to control for other variables and determine the optimal daily amount and duration of fresh moringa leaf consumption during pregnancy for improved outcomes. Additionally, efforts should focus on enhancing dietary counseling to improve hemoglobin levels, fetal and maternal nutrition, strengthening family planning services, and increasing accessibility to maternal health care to better support newborn birth weight.

## AUTHOR CONTRIBUTIONS

**Zeritu Dewana Derbo:** Conceptualization (equal); data curation (equal); formal analysis (equal); funding acquisition (equal); investigation (equal); methodology (equal); project administration (equal); resources (equal); software (equal); supervision (equal); validation (equal); visualization (equal); writing – original draft (equal); writing – review and editing (equal). **Gurmesa Tura Debelew:** Conceptualization (equal); data curation (equal); formal analysis (equal); funding acquisition (equal); investigation (equal); methodology (equal); project administration (equal); resources (equal); software (equal); supervision (equal); validation (equal); visualization (equal); writing – original draft (equal); writing – review and editing (equal).

## FUNDING INFORMATION

This work was supported by Jimma University, Ethiopia. The funder had no role in study design, data collection, analysis, preparing, and submitting the manuscript.

## CONFLICT OF INTEREST STATEMENT

The authors declare that they have no competing interests.

## Data Availability

All relevant data are within the paper. However, if additional information is required, it will be provided upon request from corresponding author.
